# Announcing the affiliation between *BMC Complementary and Alternative Medicine* and Cochrane Complementary Medicine

**DOI:** 10.1186/s12906-019-2480-1

**Published:** 2019-03-29

**Authors:** L. Susan Wieland, Karen Pilkington, Liam J. Messin

**Affiliations:** 1 Cochrane Complementary Medicine, Center for Integrative Medicine, University of Maryland School of Medicine, Baltimore, USA; 2 School of Health Sciences and Social Work, University of Portsmouth, Portsmouth, UK; 3 BMC Complementary and Alternative Medicine, London, UK

We are pleased to announce the formation of an affiliation between *BMC Complementary and Alternative Medicine* and Cochrane Complementary Medicine. This relationship has been fostered with the aim of improving the rigor and transparency of Systematic Reviews published within the journal.

Systematic reviews are considered to be the least biased method to identify, evaluate, and summarize the evidence on the effectiveness of an intervention [1]. Systematic reviews both sum up the available research and identify any outstanding gaps in the evidence, and this overall assessment then contributes to evidence-based health-care decisions made by patients, clinicians and policy makers. This is true for complementary and alternative therapies as well as for pharmaceutical drugs and other conventional therapies.

The number of published systematic reviews has increased dramatically over the last twenty years [2]. *BMC Complementary and Alternative Medicine* alone has published dozens since the journal was first established. To be of value, however, systematic reviews should be rigorously conducted and reported: poorly conducted and poorly reported systematic reviews may be harmful rather than helpful to the enterprise of summarizing the best evidence on interventions, and such reviews are a waste of limited research resources [3].

Cochrane, a non-profit organization established on the basis of an international collaboration of health researchers and other contributors, has been at the forefront of systematic review methodology and production since the organization was founded in 1993 [4]. The Cochrane Complementary Medicine Field (cam.cochrane.org) was established in 1996 to support and promote Cochrane systematic reviews of complementary, alternative, and integrative interventions. *BMC Complementary and Alternative Medicine’s* partnership with the Cochrane Complementary Medicine Field aims to strengthen the editorial processes for systematic reviews at *BMC Complementary and Alternative Medicine*, and ultimately to improve the reporting and the quality of systematic reviews published in the journal.

A systematic review is defined as “a review of the scientific evidence which applies strategies that limit bias in the assembly, critical appraisal, and synthesis of all relevant studies on the specific topic” [5]. Pre-specification of the research question and review methods are the primary instruments for reducing bias [6], therefore while quality of conduct and quality of reporting are not identical concepts they are linked and transparency and completeness in reporting review methods is critical [7]. The planning and conduct of the review should be reported in such a way that anyone reading the report can assess the strengths and weaknesses of the systematic review methods [8]. Since being developed 10 years ago, PRISMA (Preferred Reporting Items of Systematic reviews and Meta-Analyses) has become the widely accepted standard for reporting of systematic reviews [7, 9].

Beginning in April of this year *BMC Complementary and Alternative Medicine* expects all authors submitting manuscripts of systematic reviews to adhere to the following criteria:

- To present a clear research question (population, intervention, comparator, outcome(s)) for the review, and associated eligibility criteria, that includes study design, for including studies in the review;
- To have searched at least one electronic database and reported the search strategy for that database;
- To have assessed the included studies for quality or risk of bias, and used the results of this assessment to inform the conclusions of the review; and
- To complete the most recent PRISMA checklist (www.prisma-statement.org) for the above elements as well as all other items on the checklist. Where applicable authors will be required to follow and submit extended PRISMA statements (see www.prisma-statement.org/Extensions/)

Assessment against these criteria will form part of the initial editorial process for all systematic reviews that are submitted to the journal. Authors will now be required to submit a PRISMA checklist upon submission of systematic reviews and meta-analyses to *BMC Complementary and Alternative medicine.* The completion of a PRISMA checklist will aid authors in ensuring that their manuscript meets the reporting requirements, and the submission of a completed PRISMA checklist together with the manuscript will speed the checking of the manuscript, and progression to peer review.

To facilitate and maintain standards of systematic reviews at the journal the Editorial Board for *BMC Complementary and Alternative Medicine* is being expanded with the creation of a new role, Systematic Review Editor. Systematic Review Editors are members of the Cochrane Complementary Medicine Field and their sole responsibility will be initial assessment of submitted systematic review manuscripts.

The intentions with this initiative are two-fold: to establish clear criteria against which an initial assessment of systematic reviews can be carried out and to streamline and increase the efficiency of the journal’s editorial process. We hope you will join us on our journey to ensure that systematic reviews in the complementary and alternative medicine field meet the highest standards of conduct and reporting and that those that do so are published expediently.

## Abbreviations

PRISMA: Preferred Reporting Items of Systematic reviews and Meta-Analyses
